# Effect of early serum phosphate disorder on in-hospital and 28-day mortality in sepsis patients: a retrospective study based on MIMIC-IV database

**DOI:** 10.1186/s12911-024-02462-x

**Published:** 2024-02-26

**Authors:** Yinghao Luo, Yahui Peng, Yujia Tang, Pengfei Huang, Qianqian Zhang, Chunying Wang, Weiting Zhang, Jing Zhou, Longyu Liang, YuXin Zhang, Kaijiang Yu, Changsong Wang

**Affiliations:** 1 Departments of Critical Care Medicine, the First Affiliated Hospital of Harbin Medical University, 150001 Harbin, Heilongjiang China; 2 Heilongjiang Provincial Key Laboratory of Critical Care Medicine, 23 Postal Street, Nangang District, 150001 Harbin, Heilongjiang China

**Keywords:** Sepsis, Serum phosphate, MIMIC-IV, Mortality

## Abstract

**Background:**

This study aims to assess the influence of early serum phosphate fluctuation on the short-term prognosis of sepsis patients.

**Methods:**

This retrospective study used the Medical Information Mart for Intensive Care IV database to analyze serum phosphate levels in sepsis patients within 3 days of ICU admission. According to the absolute value of delta serum phosphate (the maximum value minus the minimum value of serum phosphorus measured within three days), the patients were divided into four groups, 0–1.3, 1.4–2.0, 2.1–3.1, and ≥ 3.2 mg/dl. Meanwhile, the direction of delta serum phosphate was compared. With the serum phosphate change group of 0–1.3 mg/dl as the reference group, the relationship between delta serum phosphate and in-hospital mortality and 28-day mortality was analyzed by multivariate Logistics regression analysis.

**Results:**

The study involved 1375 sepsis patients. Serum phosphate changes (0–1.3, 1.4–2.0, 2.1–3.1, and ≥ 3.2 mg/dl) correlated with in-hospital and 28-day mortality variations (*p* = 0.005, *p* = 0.008). Much higher serum phosphate fluctuation elevated in-hospital and 28-day mortality. Compared to the 0–1.3 mg/dl change group, adjusted odds ratios (OR) in other groups for in-hospital mortality were 1.25 (0.86–1.81), 1.28 (0.88–1.86), and 1.63 (1.10–2.43), and for 28-day mortality were 1.21 (0.86–1.72), 1.10 (0.77–1.57), and 1.49 (1.03–2.19). Under the trend of increasing serum phosphate, the ORs of in-hospital mortality and 28-day mortality in ≥ 3.2 mg/dl group were 2.52 and 2.01, respectively.

**Conclusion:**

In conclude, the delta serum phosphate ≥ 3.2 mg/dl was associated with in-hospital mortality and 28-day mortality in patients with sepsis.

**Supplementary Information:**

The online version contains supplementary material available at 10.1186/s12911-024-02462-x.

## Introduction

As an indispensable element of the human body, phosphate plays an important role in maintaining the integrity of cell membrane, cell signal transduction and other physiological functions [1, 2], so it is essential to maintain an appropriate phosphate concentration. The delicate balance of phosphate levels is sustained through renal clearance, intestinal absorption, and storage in the bone [3]. In critically ill patients, the meticulous balance of phosphate homeostasis often falls into neglect, yielding a susceptibility to phosphate derangements. The prevalence of hypophosphatemia and hyperphosphatemia in critically ill patients has been reported to be 20% and 45%, respectively [4]. Moreover, these perturbations in phosphate levels wield a profound impact on the prognosis of critically ill patients, particularly those subjected to mechanical ventilation, thereby augmenting hospitalization duration and mechanical ventilation duration [5].

Sepsis, as one of the common critical diseases in intensive care unit (ICU), is a life-threatening organ dysfunction resulting from an aberrant host response to infection [6], distinguished by a notable incidence and an elevated mortality rate. As the trajectory of sepsis unfolds, a myriad of immunologic and metabolic alterations incited by the host’s reaction to the pathogen come into play, exerting a discernible influence upon patient survival. Studies have shown that about 80% of patients with sepsis are accompanied by hypophosphatemia and are related to high levels of inflammatory factors such as TNF-a and IL-6 [7]. However, the correlation between serum phosphate disorders and the clinical outcome of sepsis is still controversial. In a study conducted by Shore et al., the presence of severe hypophosphatemia during sepsis imparted an alarming eightfold surge in the risk of mortality, although there were 55 patients included in the study [8]. However, several other studies have shown that hyperphosphatemia or elevated serum phosphate within the normal range is associated with higher mortality [9, 10], and serum phosphate can be used as indicators to predict the prognosis of sepsis [11]. Nevertheless, the specific serum phosphate threshold efficaciously prognosticating sepsis remains undetermined. As gleaned from antecedent studies, the intricate interplay between sepsis and serum phosphate metabolism disturbs the homeostasis of serum phosphate. The relationship between serum phosphate and patient prognosis is complicated, related not only to serum phosphate levels, but also to phosphate fluctuation. Indeed, amplified fluctuations in serum phosphate concentration were discernibly associated with a concurrent elevation in in-hospital mortality rates among patients [12]. The elucidation of the intricate relationship between sepsis and the undulations in serum phosphate concentration necessitates further erudite study.

Upon reviewing the previous studies, we found that most of the studies were limited to discussing the influence of initial serum phosphate concentration on the prognosis of sepsis, with few studies on the influence of serum phosphate fluctuation and change direction on the prognosis of sepsis. We suspected that the degree and direction of serum phosphate change may impact the prognosis of sepsis patients. Therefore, we extracted the serum phosphate data of sepsis patients admitted to the ICU in the first three days from the Medical Information Mart for Intensive Care IV (MIMIC-IV) database to explore the influence on the degree and direction of serum phosphate changes on the short-term prognosis. The large sample size of MIMIC database can make the results more robust. This approach may address the gaps of related research on the impact of serum phosphate changes on the prognosis of sepsis.

## Materials and methods

### Database and patients

This retrospective analysis utilized the MIMIC-IV database (https://mimic-iv.mit.edu/), an improved version of MIMIC-III [13], containing comprehensive hospitalization data for patients admitted to the Advanced Medical Center in Boston, USA, from 2008 to 2019. The database includes demographic information, vital signs, laboratory parameters, imaging data, and clinical results.

Access to this valuable resource was granted after successful completion of the ‘Protection of Human Subjects’ training, specifically the Data or Specimens Only Research course, resulting in Certification No. 32,286,403. Approval was obtained from the MIT Institutional Review Board and the Beth Israel Deaconess Medical Center, with a waiver for informed consent.

Our study focused on MIMIC-IV ICU patients diagnosed with Sepsis based on the Sepsis 3.0 criteria [6], who had serum phosphate measurements within 72 h of admission. Exclusion criteria included: (1) patients aged < 18 years or > 80 years; (2) patients whose Sequential Organ Failure Assessment (SOFA) score < 2 or ICU stay < 3 days; (3) patients with chronic renal failure or without serum phosphate detection and vital signs within 72 h of ICU admission; (4) patients for whom the direction of delta serum phosphate could not determine and other variables were missing.

### Data collection

We collected various patient variables, including age, gender, infection site, comorbidities (septic shock, acute kidney injury, intestinal diseases), ICU and hospital stay duration, in-hospital and 28-day mortality, mechanical ventilation duration, Glasgow Coma Scale (GCS) score [14], SOFA score and Acute Physiological and Chronic Health Assessment (APACHE II) score [15], serum phosphate, albumin, creatinine, white blood cells, pH, nutritional method (enteral or parenteral), and type of vasoactive agents. Serum phosphate levels were assessed by the first measurement after ICU admission and the maximum, minimum, and mean values during the initial 3 days. Other lab results were based on mean values within the first 24 h post-ICU admission. The vasoactive agent type was determined by the vasopressor used in the first 3 days, including norepinephrine, isoproterenol, dopamine, dobutamine, and epinephrine.

The primary research index was the delta serum phosphate during the first three days of sepsis, defined as the absolute difference between the maximum and minimum serum phosphate during the first three days. Patients with sepsis were divided into four groups according to the quartile of the delta serum phosphate: 0-1.3, 1.4-2.0, 2.1–3.1, and ≥ 3.2 mg/dl [16]. To further evaluate the effect of the direction of the delta serum phosphate on the prognosis of patients with sepsis, the time relationship between the maximum and the minimum value of serum phosphate was evaluated. We defined that when the time of the maximum value of serum phosphate was earlier than the time of the minimum value, serum phosphate was considered to show a downward trend, which was recorded as negative value. When the time of the maximum value of serum phosphate was later than the time of the minimum value, it was considered that the serum phosphate showed an upward trend and was recorded as a positive value. The direction of serum phosphate change over time was divided into eight groups: ≤-3.2, -3.1 to -2.1, -2.0 to -1.4, -1.3 to 0,0 to 1.3, 1.4 to 2.0, 2.1 to 3.1, and ≥ 3.2 mg/dl.

### Primary outcome

The primary outcomes were in-hospital and 28-day mortality.

### Statistical analysis

Analysis of variance (ANOVA) and χ2 tests were used to compare the continuous and categorical variables between the groups of delta serum phosphate. Continuous variables in baseline characteristics are presented as mean ± SD or median with interquartile ranges. Categorical variables were expressed as percentages or frequencies. Kaplan-Meier curves were constructed to illustrate survival in different groups of septic patients. Logistics regression analysis was performed to report odds ratios (OR) with 95% confidence intervals (CI) for the association between delta serum phosphate and in-hospital and 28-day mortality, compared to the delta serum phosphate group of 0-1.3 mg/dl. Meanwhile, logistic regression analysis was performed to evaluate the association between the direction of delta serum phosphate and in-hospital and 28-day mortality by using the − 1.3 to 0 mg/dl as criterion group. Three different models were constructed to analyze the association of delta serum phosphorus with in-hospital and 28-day mortality, model 1 unadjusted, model 2 adjusted for sex and age, and model 3 adjusted for some clinical characteristics. Pre-specified subgroup analyses were performed according to AKI and intestinal disease status. All statistical analyses were performed using R software, version4.2.2, along with the use of MSTATA software, *p* value < 0.05 were considered statistically significant.

## Results

### Description of study population

**Fig. 1:**
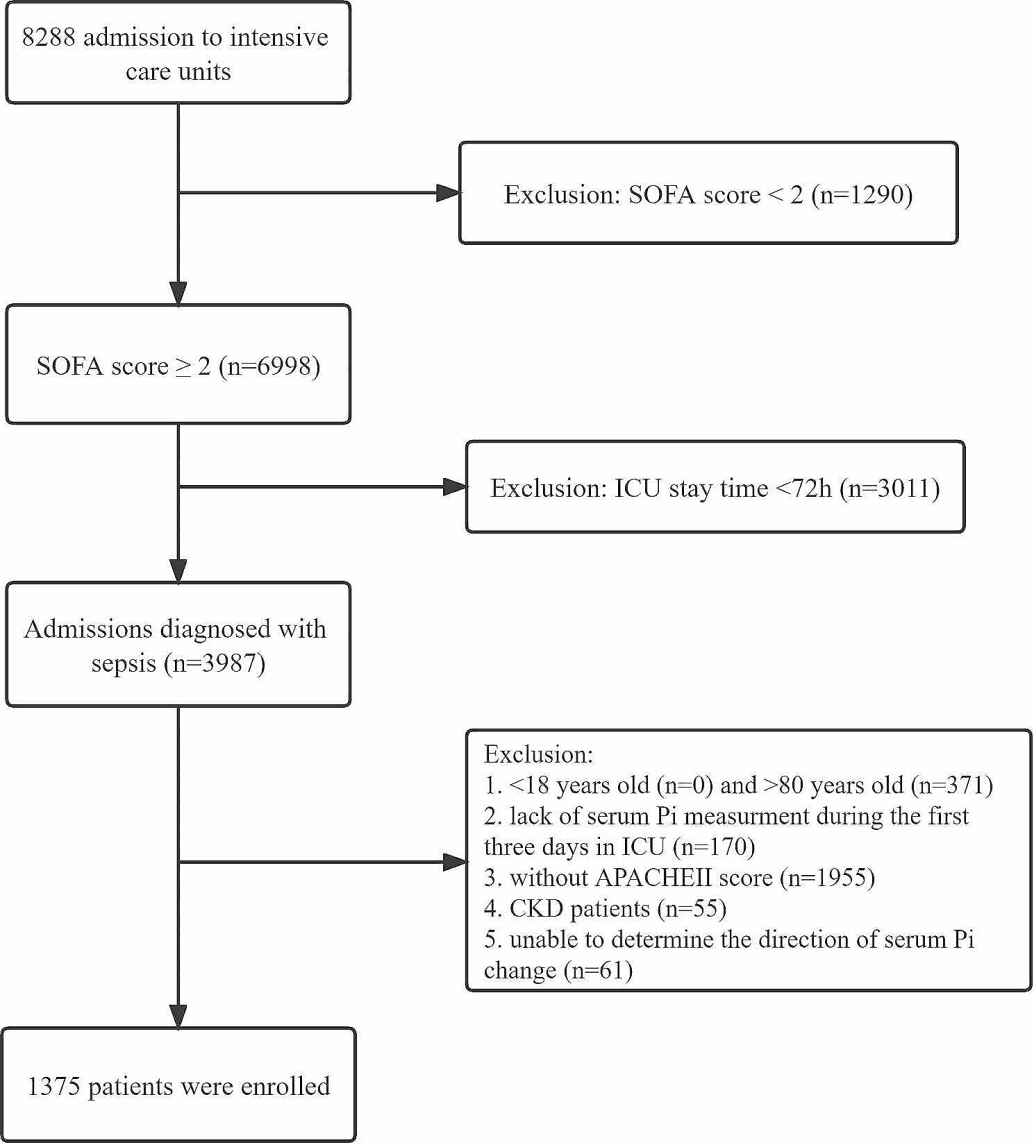
Flowchart of sepsis patients enrolled in the study. ICU: Intensive Care Unit

The flow chart showed (Fig. 1) that we extracted 8288 sepsis cases from the MIMIC-IV database, excluded some patients who did not meet the inclusion criteria, and finally a total of 1375 sepsis patients were included in the study. Supplementary Table 1 demonstrated the general characteristics of the cohort. The median age was 61, accounting for 42.04% of females. Among them, respiratory system infection, urinary system infection, soft tissue infection, digestive tract infection, and blood infection accounted for 31.78%, 2.33%, 4.15%, 17.31%, respectively. The incidence of comorbidities including AKI, intestinal disease and septic shock were 39.13%, 14.55% and 75.13%, respectively. The median SOFA and APACHE II scores of all sepsis patients were 7.92 and 28, respectively, and the median length of ICU stay and hospital stay were 8.98 days and 17.07 days, respectively.

Table 1 showed the different variables in four groups based on the quartile of the delta serum phosphate: 0-1.3 (*n* = 293), 1.4-2.0 (*n* = 355), 2.1–3.1 (*n* = 370), ≥ 3.2 mg/dl (*n* = 357). The SOFA scores in four groups were 6.80 ± 2.73, 7.29 ± 2.85, 7.79 ± 2.87 and 9.60 ± 3.29, respectively (*p* < 0.001), the APACHE II scores were 26 (23,29), 27 (23,30), 29 (23,32) and 30 (27,34), respectively (*p* < 0.001). And the in-hospital mortality in four groups were 23.89%, 28.17%, 28.11%, 36.13% (*p* = 0.005), the 28-day mortality were 31.59%, 34.37%, 32.16% and 42.02%, respectively(*p* = 0.008). In-hospital mortality and 28-day mortality almost increased with greater delta serum phosphate and more severe disease.

**Table 1:** Baseline characteristics of quartile groups of delta serum phosphate in sepsis patients

Variables	Delta serum phosphate level during the first 3 days of ICU admission (mg/dL)				P value
	0−1.3	1.4−2.0	2.1–3.1	≥ 3.2	
N	293	355	370	357	
Female, n (%)	131 (44.71)	171 (48.17)	149 (40.27)	127 (35.57)	0.005
Age, M(P25,P75)	59 (49,69)	59 (50,70)	62 (54,69)	61 (51,68)	0.030
Infection site, n (%)					0.005
-Respiratory system	110 (37.54)	118 (33.24)	112 (30.27)	97 (27.17)	
-Urinary system	7 (2.39)	10 (2.82)	8 (2.16)	7 (1.96)	
-Soft tissue infection	15 (5.12)	16 (4.51)	17 (4.60)	9 (2.52)	
-Digestive system	46 (15.70)	64 (18.03)	73 (19.73)	55 (15.41)	
-Hematologic	28 (9.56)	27 (7.60)	14 (3.78)	28 (7.84)	
-other	87 (29.69)	120 (33.80)	146 (39.46)	161 (45.10)	
Comorbidity, n (%)					
-AKI	91 (31.06)	126 (35.49)	136 (36.76)	185 (51.82)	< 0.001
-Intestine disease	35 (11.95)	52 (14.65)	73 (19.73)	40 (11.20)	0.005
-Septic shock	212 (72.35)	261 (73.52)	274 (74.05)	286 (80.11)	0.083
Hospital stay time /day, M(P25,P75)	15.81 (9.84,26.77)	16.01 (10.90,28.86)	17.88 (11.24,27.21)	17.25 (9.63,28.64)	0.557
ICU stay time /day, M(P25,P75)	8.23 (5.70,14.84)	8.09 (5.29,13.79)	8.96 (5.84,15.55)	9.64 (5.93,16.92)	0.128
Invasive mechanical ventilation time /h, M(P25,P75)	121.35 (50.00,218.00)	89.10 (33.00,182.90)	97.80 (52.00,208.13)	126.00 (67.50,233.50)	0.035
Noninvasive mechanical ventilation time /h, M(P25,P75)	16.75 (2.16,44.50)	31.08 (4.50,68.00)	26.33 (5.78,59.36)	17.80 (0,50.83)	0.161
GCS score, M(P25,P75)	7.67 (5.92,10.08)	8.39 (6.50,10.71)	8.46 (6.69,10.40)	10.13 (8.04,12.46)	0.667
SOFA score, $\bar x \pm s$	6.80 ± 2.73	7.29 ± 2.85	7.79 ± 2.87	9.60 ± 3.29	< 0.001
APACHEII score, M(P25,P75)	26 (23,29)	27 (23,30)	29 (23,32)	30 (27,34)	< 0.001
[Pi]_first_ (mg/dl), M(P25,P75)	3.20 (2.50,4.10)	3.60 (3.00,4.70)	4.20 (3.40,5.10)	5.70 (4.20,7.15)	< 0.001
[Pi]_min_ (mg/dl), M(P25,P75)	2.60 (2.10,3.50)	2.60 (2.00,3.60)	2.60 (1.95,3.45)	2.60 (1.80,3.60)	0.265
[Pi]_max_ (mg/dl), M(P25,P75)	3.50 (3.00,4.40)	4.40 (3.70,5.30)	5.20 (4.30,6.20)	7.10 (6.00,8.35)	< 0.001
[Pi]_mean_ (mg/dl), M(P25,P75)	3.03 (2.58,3.84)	3.41 (2.71,4.30)	3.74 (3.07,4.68)	4.54 (3.44,5.86)	< 0.001
Albumin (g/dl), M(P25,P75)	2.68 (2.20,3.00)	2.60 (2.30,2.90)	2.70 (2.35,3.10)	2.60 (2.30,3.13)	0.109
Creatinine (mg/dl), M(P25,P75)	1.00 (0.67,2.03)	1.37 (0.93,2.17)	1.58 (1.04,2.46)	2.52 (1.70,3.74)	< 0.001
WBC (*10^9^/), M(P25,P75)	14.35 (9.83,20.65)	13.33 (9.00,21.00)	16.25 (10.95,21.04)	16.07 (9.80,21.93)	0.546
pH, $\bar x \pm s$	7.36 ± 0.07	7.35 ± 0.07	7.32 ± 0.07	7.29 ± 0.08	< 0.001
Enteral nutrition, n(%)	102 (34.81)	100 (28.17)	115 (31.08)	98 (27.45)	0.167
Parenteral nutrition, n(%)	33 (11.26)	43 (12.11)	43 (11.62)	42 (11.76)	0.990
Types of vasoactive agents, M(P25,P75)	1 (1,2)	1 (1,2)	2 (1,2)	2 (1,2)	< 0.001
In-hospital mortality, n(%)	70 (23.89)	100 (28.17)	104 (28.11)	129 (36.13)	0.005
28-day mortality, n(%)	89 (31.59)	122 (34.37)	119 (32.16)	150 (42.02)	0.008

AKI: Acute Kidney Injure, GCS: Glasgow Coma Scale, SOFA: Sequential Organ Failure Assessment, APACHE II: Acute Physiological and Chronic Health Assessment. [Pi]_first_: The first serum phosphate measurement during the first 3 days of ICU admission; [Pi]_min_: The lowest serum phosphate measurement during the first 3 days of ICU admission; [Pi]_max_: The highest serum phosphate measurement during the first 3 days of ICU admission; [Pi]_mean_: The mean serum phosphate measurement during the first 3 days of ICU admission

### The association between serum phosphate changes and in-hospital and 28-day mortality

Logistics regression was performed to construct three different models to describe the association between delta serum phosphate and in-hospital mortality and 28-day mortality. When the delta serum phosphate was analyzed as a continuous variable, delta serum phosphate was associated with in-hospital mortality, with a 12% increased risk of in-hospital mortality per 1 mg/dl increase. However, the adjusted OR for delta serum phosphate and 28-day mortality was 1.08 (0.99,1.18), which was not statistically significant (Supplementary Table 2).

**Fig. 2:**
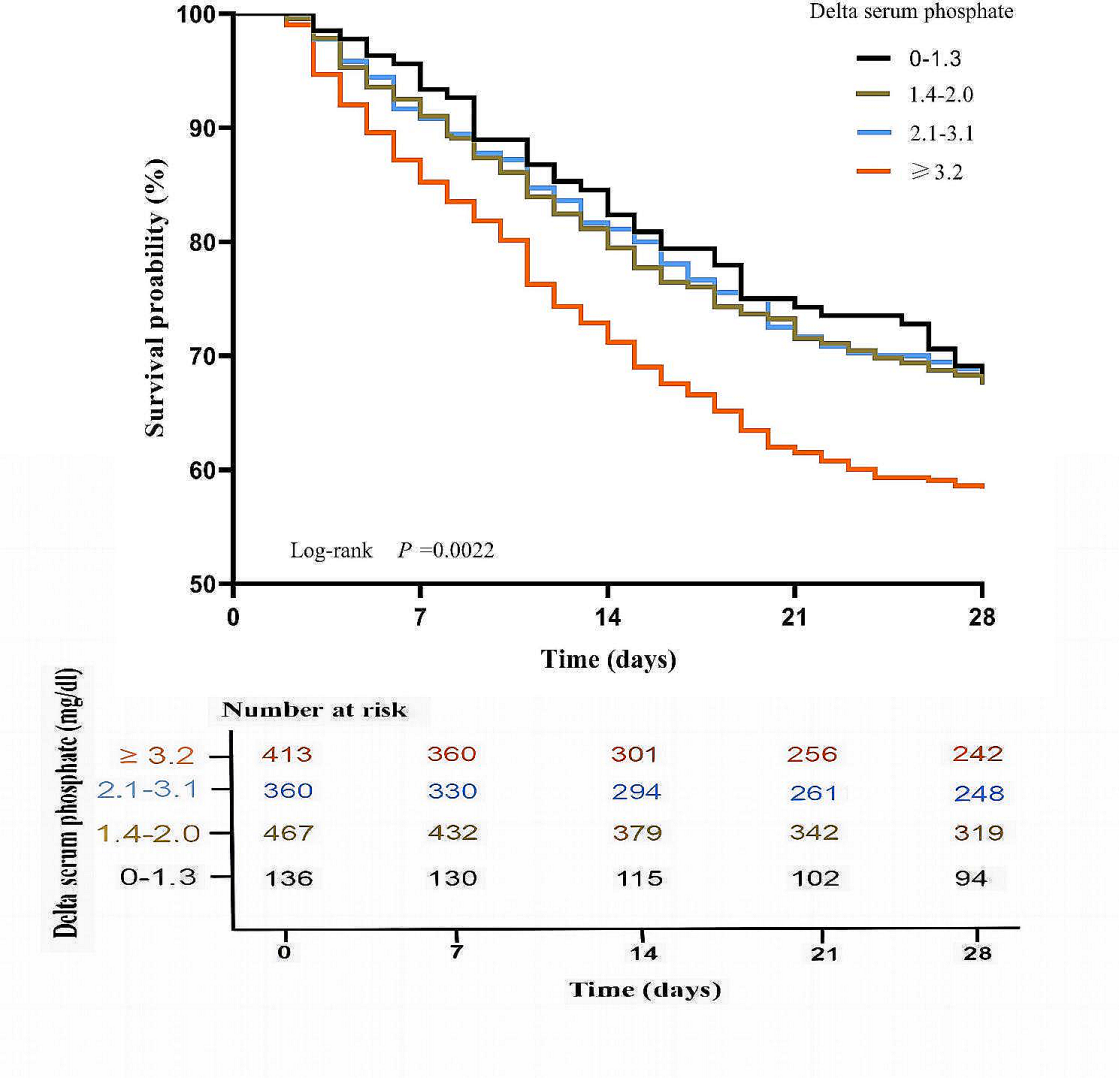
Kaplan–Meier survival analysis plots for 28-day mortality with delta serum phosphate category

We then evaluated the three models with the delta serum phosphate transformed from continuous to categorical variables. Kaplan-Meier curves were constructed to illustrate survival in different groups of septic patients. As shown in Fig. 2: 28-day mortality was significantly higher in the delta serum phosphate ≥ 3.2 mg/dl group than in the other groups (log-rank, *p* = 0.0022). In model 3, increased delta serum phosphate was associated with increased in-hospital mortality and 28-day mortality after adjustment for confounding factors. Compared with the 0-1.3 mg/dl group, the 1.4-2.0 mg/dl group, 2.1-3.1 mg/dl group, and ≥ 3.2 mg/dl group had an adjusted ORs for in-hospital mortality of 1.25 (0.86 to 1.81), 1.28 (0.88 to 1.86), and 1.63 (1.10 to 2.43), respectively; and the adjusted ORs for 28-day mortality were 1.21 (0.86 to 1.72), 1.10 (0.77 to 1.57), and 1.49 (1.03 to 2.19), respectively. *P* values were statistically significant only in the ≥ 3.2 mg/dl group (Table 2), which demonstrated that delta serum phosphate more than 3.2 mg/dl were associated with in-hospital and 28-day mortality.

**Table 2:** The association between delta serum phosphate and in-hospital mortality and 28-day mortality

Outcome	Delta serum phosphate level during the first 3 days of ICU admission (mg/dL)			
	0−1.3	1.4−2.0	2.1–3.1	≥ 3.2
In-hospital mortality, OR (95% CI, *P*)				
-Model 1, unadjusted	1.0 (Ref)	1.25 (0.88–1.78), 0.218	1.25 (0.88–1.77), 0.221	1.80 (1.28–2.55), < 0.001
-Model 2^a^	1.0 (Ref)	1.26 (0.88–1.80), 0.207	1.28 (0.90–1.82), 0.177	1.97 (1.39–2.80), < 0.001
-Model 3^b^	1.0 (Ref)	1.25 (0.86–1.81), 0.246	1.28 (0.88–1.86), 0.202	1.63 (1.10–2.43), 0.016
28-day mortality, OR (95% CI, *P*)				
-Model 1, unadjusted	1.0 (Ref)	1.20 (0.86–1.67), 0.281	1.09 (0.78–1.52), 0.622	1.66 (1.20–2.31), 0.002
-Model 2^a^	1.0 (Ref)	1.22 (0.87–1.71), 0.247	1.11 (0.79–1.55), 0.549	1.82 (1.31–2.55), < 0.001
-Model 3^b^	1.0 (Ref)	1.21 (0.86–1.72), 0.282	1.10 (0.77–1.57), 0.605	1.49 (1.03,2.19), 0.037

a: Adjusted for sex and age

b: Adjusted for sex, age, infection site, AKI, intestine disease, invasive mechanical ventilation time, APACHEII score, PH, creatinine

### The association between the direction of delta serum phosphate and in-hospital mortality and 28-day mortality

We then further explored the relationship between the direction of delta serum phosphate and in-hospital mortality and 28-day mortality. The results showed that higher in-hospital and 28-day mortality were associated with both decreasing and increasing trends in delta serum phosphate (Table 3). The highest in-hospital mortality (32.43%) and 28-day mortality (39.77%) were observed in the ≤-3.2 mg/dl group, with adjusted ORs of 1.40 (0.86 to 2.30) and 1.49 (0.94 to 2.38), respectively, although there was no statistical significance. In the upward trend of serum phosphate, the highest in-hospital mortality (45.92%) and 28-day mortality (47.96%) were also observed in the ≥ 3.2 mg/dl group, with adjusted ORs of 2.52 (1.43 to 4.46) and 2.01 (1.16 to 3.50), respectively (*p* < 0.05). It is noteworthy that the increasing risk with the rising trend of delta serum phosphate exceeds the risk of serum phosphate in decreasing trend.

**Table 3:** The association between the direction of delta serum phosphate and in-hospital mortality and 28-day mortality

Phosphate change (mg/dL)	Classification	Mortality	Model 1, OR (95% CI, P)	Model 2, OR (95% CI, P)	Model 3, OR (95% CI, P)
≤−3.2	In-hospital mortality	84 (32.43)	1.67 (1.08–2.60), 0.022	1.80 (1.16–2.82), 0.009	1.40 (0.86–2.30), 0.175
	28-day mortality	103 (39.77)	1.70 (1.13–2.58), 0.011	1.87 (1.24–2.85), 0.003	1.49 (0.94–2.38), 0.093
−3.1 to−2.1	In-hospital mortality	67 (26.17)	1.23 (0.79–1.94), 0.362	1.25 (0.80–1.97), 0.339	1.19 (0.74–1.92), 0.474
	28-day mortality	78 (30.47)	1.13 (0.74–1.73), 0.568	1.14 (0.75–1.75), 0.540	1.11 (0.71–1.74), 0.661
−2 to−1.4	In-hospital mortality	68 (31.19)	1.58 (1.00−2.49), 0.05	1.55 (0.99–2.47), 0.06	1.53 (0.96–2.47), 0.076
	28-day mortality	85 (38.99)	1.65 (1.08–2.53), 0.021	1.65 (1.08–2.55), 0.022	1.66 (1.07–2.60), 0.025
−1.3 to 0	In-hospital mortality	40 (22.35)	1.0 (Ref)	1.0 (Ref)	1.0 (Ref)
	28-day mortality	50 (27.93)	1.0 (Ref)	1.0 (Ref)	1.0 (Ref)
0 to 1.3	In-hospital mortality	30 (26.32)	1.24 (0.72–2.14), 0.438	1.23 (0.71–2.14), 0.452	1.14 (0.64–2.01), 0.657
	28-day mortality	39 (34.21)	1.34 (0.81–2.23), 0.255	1.36 (0.81–2.26), 0.242	1.30 (0.76–2.21), 0.341
1.4 to 2.0	In-hospital mortality	32 (23.52)	1.07 (0.68–1.81), 0.804	1.11 (0.65–1.90), 0.697	0.99 (0.57–1.72), 0.971
	28-day mortality	37 (27.21)	0.96 (0.58–1.59), 0.886	1.02 (0.61–1.68), 0.948	0.92 (0.54–1.56), 0.760
2.1 to 3.1	In-hospital mortality	37 (32.17)	1.65 (0.97–2.79), 0.063	1.72 (1.01–2.94), 0.044	1.63 (0.94–2.83), 0.084
	28-day mortality	41 (35.65)	1.43 (0.86–2.36), 0.163	1.50 (0.90–2.50), 0.116	1.43 (0.84–2.43), 0.190
≥ 3.2	In-hospital mortality	45 (45.92)	2.95 (1.74–5.04), < 0.001	3.31 (1.94–5.70), < 0.001	2.52 (1.43–4.46), 0.001
	28-day mortality	47 (47.96)	2.38 (1.43–3.99), < 0.001	2.63 (1.56–4.45), < 0.001	2.01 (1.16–3.50), 0.013

Model 1: Unadjusted

Model 2: Adjusted for sex and age

Model 3: Adjusted for sex, age, infection site, AKI, intestine disease, invasive mechanical ventilation time, APACHEII score, PH, creatinine

### Subgroup analysis based on AKI and intestinal disease

A total of 538 patients (39.13%) presented with AKI. Subgroup analysis showed: Higher in-hospital mortality and 28-day mortality were detected at delta serum phosphate ≥ 3.2 mg/dl group in AKI and non-AKI sepsis patients (39.46% and 32.56%, respectively). Interestingly, there was no significant difference in the association between delta serum phosphate ≥ 3.2 mg/dl group and in-hospital mortality and 28-day mortality in AKI patients, whereas in non-AKI patients, delta serum phosphate 2.1–3.1 mg/dl group demonstrated a statistically significant difference in in-hospital mortality [OR 1.70 (1.04, 2.81), *p* = 0.035]. This seemd to indicate that the greater fluctuation of serum phosphate was related to the increased risk of death of patients. In patients with AKI, the fluctuation of serum phosphate may be the direct result of AKI, and the relationship with mortality may be affected by other factors. In contrast, it was statistically significant in non-AKI patients, indicating that moderate serum phosphate fluctuation may be a poor prognosis factor after excluding the influence of AKI. It also showed that delta phosphate was related to the risk of death and may be used as a prognostic indicator. (Table 4).

**Table 4:** Subgroup analysis based on acute kidney renal disease (AKI) status

Outcome	Delta serum phosphate level during the first 3 days of ICU admission (mg/dL)			
	0−1.3	1.4−2.0	2.1–3.1	≥ 3.2
**AKI during hospitalization(***n* **=** **538)**				
N	91	126	136	185
In-hospital mortality	32 (35.16)	41 (32.54)	37 (27.21)	73 (39.46)
Mortality, OR (95% CI, *P*)				
-Model 1, unadjusted	1.0 (Ref)	0.89 (0.50,1.58), 0.686	0.69 (0.39,1.22), 0.202	1.20 (0.72,2.04), 0.490
-Model 2^a^	1.0 (Ref)	0.93 (0.52,1.65), 0.797	0.71 (0.40,1.26), 0.237	1.35 (0.80,2.31), 0.272
-Model 3^b^	1.0 (Ref)	0.99 (0.55,1.80), 0.969	0.80 (0.44,1.48), 0.479	1.47 (0.81,2.67), 0.206
28-day mortality	39 (42.86)	47 (37.30)	41 (30.15)	84 (45.41)
Mortality, OR (95% CI, *P*)				
-Model 1, unadjusted	1.0 (Ref)	0.79 (0.46,1.38), 0.409	0.58 (0.33,1.00), 0.05	1.11 (0.67,1.85), 0.689
-Model 2^a^	1.0 (Ref)	0.83 (0.48,1.44), 0.506	0.58 (0.33,1.02), 0.057	1.21 (0.72,2.03), 0.471
-Model 3^b^	1.0 (Ref)	0.91 (0.51,1.61), 0.736	0.66 (0.37,1.19), 0.168	1.30 (0.74,2.32), 0.363
**No AKI during hospitalization(***n* **=** **837)**				
N	202	229	234	172
In-hospital mortality	38 (18.81)	59 (25.76)	67 (28.63)	56 (32.56)
Mortality, OR (95% CI, *P*)				
-Model 1, unadjusted	1.0 (Ref)	1.50 (0.95,2.39), 0.086	1.73 (1.11, 2.74), 0.017	2.08 (1.30,3.37), 0.002
-Model 2^a^	1.0 (Ref)	1.49 (0.94,2.38), 0.094	1.78 (1.13,2.83), 0.013	2.26 (1.40,3.68), < 0.001
-Model 3^b^	1.0 (Ref)	1.43 (0.88,2.34), 0.148	1.70 (1.04,2.81), 0.035	1.65 (0.95,2.90), 0.078
28-day mortality	50 (24.75)	75 (32.75)	78 (33.33)	66 (38.37)
Mortality, OR (95% CI, *P*)				
-Model 1, unadjusted	1.0 (Ref)	1.48 (0.97,2.27), 0.069	1.52 (1.00,2.32), 0.050	1.89 (1.22,2.96), 0.005
-Model 2^a^	1.0 (Ref)	1.48 (0.97,2.28), 0.073	1.57 (1.03,2.42), 0.038	2.11 (1.34,3.33), 0.001
-Model 3^b^	1.0 (Ref)	1.40 (0.90,2.20), 0.142	1.47 (0.93,2.35), 0.101	1.48 (0.87,2.51), 0.146

a: Adjusted for sex and age

b: Adjusted for sex, age, infection site, intestine disease, invasive mechanical ventilation time, APACHEII score, PH, creatinine

Similarly, in the subgroup analysis of intestinal disease, 14.55% of patients had intestinal disease. In patients with and without intestinal disease, the higher in-hospital mortality and 28-day mortality (32.50% and 36.59%, respectively) was detected in serum phosphate change ≥ 3.2 mg/dl. There was no significant difference between delta serum phosphate and in-hospital and 28-day mortality in patients with intestinal disease, whereas in patients without intestinal disease, the delta serum phosphate ≥ 3.2 mg/dl group demonstrated a statistically significant difference in in-hospital mortality [OR, 1.54(1.02,2.36), *p* = 0.043]. This indicated that the greater delta serum phosphate was a possible risk factor for mortality. In patients with intestinal diseases, it may affect the absorption and metabolism of phosphate to some extent, but the change of serum phosphate may not the main cause of dominant mortality risk, which may be caused by the severity of intestinal disorders and other complications. (Table 5).

**Table 5:** Subgroup analysis based on intestine disease status

Outcome	Delta serum phosphate level during the first 3 days of ICU admission (mg/dL)			
	0−1.3	1.4−2.0	2.1–3.1	≥ 3.2
**Intestine disease during hospitalization(***n* **=** **200)**				
N	35	52	73	40
In-hospital mortality	5 (14.29)	9 (17.31)	19 (26.03)	13 (32.50)
Mortality, OR (95% CI, *P*)				
-Model 1, unadjusted	1.0 (Ref)	1.26 (0.39,4.43), 0.707	2.11 (0.76,6.88), 0.176	2.89 (0.95,9.99), 0.072
-Model 2^a^	1.0 (Ref)	1.01 (0.31,3.64), 0.989	2.20 (0.77,7.28), 0.161	3.41 (1.09,12.2), 0.043
-Model 3^b^	1.0 (Ref)	0.87 (0.25,3.25), 0.826	1.91 (0.63,6.65), 0.271	2.87 (0.84,11.0), 0.104
28-day mortality	9 (25.71)	10 (19.23)	22 (30.14)	18 (45.00)
Mortality, OR (95% CI, *P*)				
-Model 1, unadjusted	1.0 (Ref)	0.69 (0.25,1.94), 0.474	1.25 (0.51,3.20), 0.635	2.36 (0.90,6.52), 0.086
-Model 2^a^	1.0 (Ref)	0.57 (0.20,1.65), 0.296	1.24 (0.50,3.23), 0.645	2.71 (1.01,7.69), 0.052
-Model 3^b^	1.0 (Ref)	0.60 (0.19,1.86), 0.368	1.24 (0.46,3.54), 0.677	2.94 (0.97,9.50), 0.061
**No intestine disease during hospitalization(***n* **=** **1175)**				
N	258	303	297	317
In-hospital mortality	65 (25.19)	91 (30.03)	85 (28.62)	116 (36.59)
Mortality, OR (95% CI, *P*)				
-Model 1, unadjusted	1.0 (Ref)	1.27 (0.88,1.86), 0.203	1.19 (0.82,1.74), 0.365	1.71 (1.20,2.47), 0.004
-Model 2^a^	1.0 (Ref)	1.31 (0.90,1.92), 0.156	1.23 (0.84,1.80), 0.289	1.87 (1.29,2.70), < 0.001
-Model 3^b^	1.0 (Ref)	1.29 (0.88,1.92), 0.196	1.19 (0.80,1.79), 0.386	1.54 (1.02,2.36), 0.043
28-day mortality	80 (31.01)	112 (36.96)	97 (32.66)	132 (41.64)
Mortality, OR (95% CI, *P*)				
-Model 1, unadjusted	1.0 (Ref)	1.30 (0.92,1.86), 0.139	1.08 (0.75,1.55), 0.677	1.59 (1.13,2.25), 0.009
-Model 2^a^	1.0 (Ref)	1.36 (0.95,1.95), 0.092	1.11 (0.77,1.60), 0.564	1.74 (1.23,2.49), 0.002
-Model 3^b^	1.0 (Ref)	1.32 (0.91,1.91), 0.146	1.04 (0.71,1.54), 0.825	1.36 (0.91,2.04), 0.136

a: Adjusted for sex and age

b: Adjusted for sex, age, infection site, AKI, invasive mechanical ventilation time, APACHEII score, PH, creatinine

## Discussion

In our study, a major finding was revealed. When serum phosphate concentration elevation ≥ 3.2 mg/dl, the risk of in-hospital and 28-day mortality increased by 63% and 49% respectively. As far as we know, this is the first research to study the relationship between early serum phosphate changes and early prognosis in a large sample of sepsis patients.

Phosphate is present in all cells of the body and is involved in various physiological activities, such as nucleic acid synthesis [17], cell signal transduction [18], maintaining membrane integrity and enzyme activity [19]. Researches have shown that maintaining a healthy balance of serum phosphate is crucial for good health. Both hypophosphatemia and hyperphosphatemia in the body have been linked to negative impacts and poor prognosis [8, 10]. In a cross-sectional study, Haider et al. noted that hyperphosphatemia is an independent risk factor for death and patients with hyperphosphatemia had longer hospital stays than those with normal phosphate or hypophosphatemia, while hypophosphatemia was not associated with mortality [20]. Another study observed patients with bloodstream infection in ICU, and the 90-day mortality rate was found to be almost twofold higher in patients with bloodstream infection who had hypophosphatemia [21]. Current studies mainly focus on baseline phosphate levels, but emerging evidence suggests that even subtle fluctuations within the normal range can increase the risk of mortality in patients with sepsis [10]. Xu X et al. detected higher mortality in patients with fluctuating serum phosphate levels by comparing patients with fluctuating serum phosphate levels with those with stable serum phosphate levels [22]. In addition, Kim et al. [23] showed that higher serum phosphate fluctuations are associated with higher hospital mortality. Compared with the initial serum phosphate measurement, the maximum-minimum phosphate difference (ΔP) has a better predictive ability, which indicates that the fluctuation of serum phosphate was also an important factor affecting the prognosis. Reflecting upon our own findings, the discernment of elevated in-hospital and 28-day mortality rates within the cohort characterized by serum phosphate elevation ≥ 3.2 mg/dl substantiates its role as a harbinger of untoward early outcomes among sepsis patients, thus corroborating the aforementioned observations. In a recent study, the correlation between delta serum phosphate and the prognosis of patients with sepsis was also studied, and a non-linear relationship between delta serum phosphate and 28-day mortality was found, with similar results as ours [24]. The delta serum phosphate in this study was calculated as the difference between the initial and final serum phosphate levels measured in the ICU. Due to the variety of treatments that patients with sepsis receive in the ICU, it may not capture true fluctuations in serum phosphate and may not reflect the actual relationship between serum phosphate increments and mortality in patients with sepsis. Therefore, we selected the absolute difference between the maximum and minimum serum phosphate during the first three days of ICU admission, which might better reflect the real fluctuation of serum phosphate to some extent. At the same time, we further explored the influence of the direction of delta serum phosphate on the prognosis of sepsis patients, in order to more comprehensively explore the influence of delta serum phosphate on patients.

The mechanism by which elevated serum phosphate leads to poor prognosis is unclear. Intestinal absorption and renal clearance seem to play important roles in serum phosphate homeostasis. However, in our results significant serum phosphate changes were associated with poor early prognosis, independent of AKI and intestinal disease, which indicated that serum phosphate can affect the prognosis independently of renal and intestinal factors.Previous studies have shown that elevated serum phosphate levels are associated with cardiovascular disease [25], vascular calcification [26], reduced muscle strength [27], and the toxic effects of phosphate [28]. Elevated serum phosphate levels have the potential to heighten the risk of cardiovascular disease and microcirculatory dysfunction through inflammatory responses [25], oxidative stress, vascular calcification, endothelial dysfunction, and other intricate mechanisms. The context of sepsis often engenders ischemic injury and cellular damage, leading to the liberation of phosphate and consequent elevation of serum phosphate levels [29]. Simultaneously, heightened serum phosphate levels can impair mitochondrial function, amplifying the production of reactive oxygen species, curtailing the phosphorylation of endothelial nitric oxide synthase, and dampening nitric oxide production. These cascading effects further exacerbate microcirculatory disturbances and ischemic injuries [28]. In our study, patients with large phosphate changes required more vasoactive drugs, which may be related to vascular disease caused by elevated serum phosphorus. In addition, elevated serum phosphate may increase the risk of calcium phosphate precipitation and cause vascular calcification, leading to hypocalcemia, which can lead to life-threatening arrhythmias and cardiac arrest [30].

Relevant studies have underscored the potential consequences of serum phosphate decrease or depletion, including compromised myocardial energy metabolism and reduced myocardial contractility [31], and hypophosphatemia caused by the decrease of serum phosphate in the early stage of sepsis is related to arrhythmia [32]. Another study showed that severe hypophosphatemia can predict an almost eight-fold increase in mortality in patients with sepsis [8]. However, we found that the degree of serum phosphate decline in patients with sepsis does not seem to be the risk factor for early prognosis, and it is not clear whether the trend of serum phosphate decline can predict mortality or is merely a marker of disease severity.

This is a large retrospective study based on the MIMIC database, and in our study, the risk of in-hospital mortality and 28-day mortality were increased by 63% and 49%, respectively, when delta serum phosphate was ≥ 3.2 mg/dl. However, due to the limitations of the MIMIC database, there were some confounding factors that were not documented and included in the study. Although we tried to take into account as many factors as possible that may influence serum phosphate fluctuations, such as AKI and intestinal disease comorbidities, nutritional patterns, etc., it is not clear whether the medications, other therapeutic measures, and dietary habits received by the patients have an impact on the fluctuation of serum phosphate. In addition, although the effects of AKI and intestinal disease on serum phosphate were considered, the relationship between AKI classification and intestinal disease severity affecting serum phosphate fluctuations and mortality was unknown. This may have implications for the comprehensiveness of the interpretation of the results and needs to be explored in greater depth in future studies. Overall, our results highlight the importance of serum phosphate fluctuations on the prognosis of septic patients, which may be an important guide for clinical decision-making and patient management.

Our results revealed a 63% and 49% increased risk of in-hospital mortality and 28-day mortality, respectively, in patients with sepsis when delta serum phosphate concentrations were ≥ 3.2 mg/dl, prompting us to consider how this can be applied in clinical practice and future research directions. In clinical work, serum phosphate seems to be a simple and easily accessible biochemical indicator to be included in the evaluation and monitoring of sepsis patients, so as to more accurately judge the prognostic risk of patients. For patients with elevated serum phosphate, some clinical measures (such as interventions targeting phosphate metabolism) can be used to adjust the serum phosphate balance to reduce mortality. At the same time, the association between increased serum phosphate and increased mortality in patients with sepsis warrants further molecular mechanism studies to explore the biological causes, which may lead to new therapeutic targets or interventions. It may be considered to combine serum phosphate concentration with other clinical parameters (such as inflammatory indicators, organ function, etc.) to establish a more complete prognostic assessment model and improve the ability to identify prognostic risk in patients with sepsis.

The strength of our study lies in its comprehensive scope, including a large cohort of sepsis patients from the MIMIC-IV database. We carefully adjusted for potential confounding variables to establish the independent prognostic significance of serum phosphate elevation (≥ 3.2 mg/dl) for early outcomes. However, there are limitations to consider. Firstly, important clinical details such as interventions for hypophosphatemia or hyperphosphatemia, use of phosphate preparations, dietary inorganic phosphorus food, and hormones impacting phosphate homeostasis were not captured, which may affect the real situation of serum phosphate and bias the results. Secondly, this study is a retrospective study, which will inevitably be affected by the accuracy or completeness of data records. The missing of relevant data and unmeasured variables may have a certain impact on the prognosis and have potential bias on the results. Thirdly, we determined the delta serum phosphate direction based on zenith and nadir values within the first three days of sepsis onset, but variability in phosphate measurement intervals across patients may affect the precision of assessing serum phosphate fluctuation. Finally, our study is based on the cohort of sepsis patients in MIMIC-IV database, and the results reflect the relationship between serum phosphate fluctuation and sepsis in this group. Whether it can be extended to other regions or people needs more prospective research to confirm our results.

## Conclusion

In conclusion, we found that serum phosphate fluctuation in the early stages of sepsis were associated with poor prognosis, and that delta serum phosphate ≥ 3.2 mg/dl were associated with in-hospital mortality and 28-day mortality in patients with sepsis.

### Electronic supplementary material

Below is the link to the electronic supplementary material.

Supplementary Material 1

Supplementary Material 2

## Data Availability

No datasets were generated or analysed during the current study.

## Abbreviations

MIMIC-IV: Medical Information Mart for Intensive Care IV
ICU: Intensive care unit
SOFA: Sequential Organ Failure Assessment
AKI: Acute kidney injure
GCS: Glasgow Coma Scale
APACHE II: Acute Physiological and Chronic Health Assessment
ORs: Odds ratios
CI: Confidence intervals
