# Supplementary material for: Effect of early serum phosphate disorder on in-hospital and 28-day mortality in sepsis patients: a retrospective study based on MIMIC-IV database
Source: BMC Med Inform Decis Mak. 2024 Feb 26;24:59. doi: 10.1186/s12911-024-02462-x (PMC10898106; doi:10.1186/s12911-024-02462-x)
Supplement: Supplementary file 1 — Supplementary Material 1 [file 12911_2024_2462_MOESM1_ESM.docx]

Supplementary Table 1: Basic characteristics of sepsis patients

| Variables |  |
| --- | --- |
| N | 1375 |
| Female, n (%) | 578 (42.04) |
| Age, M(P25,P75) | 61 (51,69) |
| Infection site, n (%) |  |
| -Respiratory system | 437 (31.78) |
| -Urinary system | 32 (2.33) |
| -Soft tissue infection | 57 (4.15) |
| -Digestive system | 238 (17.31) |
| -Hematologic | 97 (7.05) |
| -other | 514 (37.38) |
| **Comorbidity**, n (%) |  |
| -AKI | 538 (39.13) |
| -Intestine disease | 200 (14.55) |
| -Septic shock | 1033 (75.13) |
| Hospital stay time /day, M(P25,P75) | 17.07 (10.65,28.05) |
| ICU stay time /day, M(P25,P75) | 8.98 (5.69,15.18) |
| Invasive mechanical ventilation time /h, M(P25,P75) | 108.70 (51.70,211.60) |
| Noninvasive mechanical ventilation time /h, M(P25,P75) | 24.00 (0.51,55.00) |
| GCS score, M(P25,P75) | 15.00 (14.71,15.00) |
| SOFA score, $\overline{\text{x}}\pm\text{s}$ | 7.92 ± 3.13 |
| APACHEII score, M(P25,P75) | 28 (24,32) |
| [Pi]_first_ (mg/dl), M(P25,P75) | 4.20 (3.20,5.60) |
| [Pi]_min_ (mg/dl), M(P25,P75) | 2.60 (1.90,3.50) |
| [Pi]_max_ (mg/dl), M(P25,P75) | 5.20 (4.00,6.60) |
| [Pi]_mean_ (mg/dl), M(P25,P75) | 3.67 (2.97,4.99) |
| Albumin (g/dl), M(P25,P75) | 2.60 (2.30,3.10) |
| Creatinine (mg/dl), M(P25,P75) | 1.70 (1.00,2.80) |
| Wbc (*10^9^/), M(P25,P75) | 15.10 (9.75,21.05) |
| PH, $\overline{\text{x}}\pm\text{s}$ | 7.33 ± 0.08 |
| Enteral nutrition, n(%) | 415 (30.18) |
| Parenteral nutrition, n(%) | 161 (11.71) |
| Types of vasoactive drugs, M(P25,P75) | 2 (1,2) |
| Hospital mortality, n(%) | 403 (29.31) |
| 28-day mortality, n(%) | 480 (34.91) |

AKI: Acute Kidney Injure, GCS: Glasgow Coma Scale, SOFA: Sequential Organ Failure Assessment, APACHE II: Acute Physiological and Chronic Health Assessment. [Pi]_first_: The first serum phosphate measurement during the first 3 days of ICU admission; [Pi]_min_: The lowest serum phosphate measurement during the first 3 days of ICU admission; [Pi]_max_: The highest serum phosphate measurement during the first 3 days of ICU admission; [Pi]_mean_: The mean serum phosphate measurement during the first 3 days of ICU admission.
