# Supplementary material for: Effect of early serum phosphate disorder on in-hospital and 28-day mortality in sepsis patients: a retrospective study based on MIMIC-IV database
Source: BMC Med Inform Decis Mak. 2024 Feb 26;24:59. doi: 10.1186/s12911-024-02462-x (PMC10898106; doi:10.1186/s12911-024-02462-x)
Supplement: Supplementary file 2 — Supplementary Material 2 [file 12911_2024_2462_MOESM2_ESM.docx]

Supplementary Table 2: The association between serum phosphate changes (as continuous variable) and in-hospital mortality and 28-day mortality.

| Outcome | Changes in serum phosphate level during the first 3 days of ICU admission (mg/dL) |
| --- | --- |
| Hospital mortality, OR (95% CI, P) |  |
| -Model 1, unadjusted | 1.15 (1.07,1.24), 0.001 |
| -Model 2a | 1.18 (1.09,1.27), 0.001 |
| -Model 3b | 1.12 (1.02,1.22), 0.013 |
| 28-day mortality, OR (95% CI, P) |  |
| -Model 1, unadjusted | 1.12 (1.04,1.21), 0.002 |
| -Model 2a | 1.15 (1.07,1.24), 0.001 |
| -Model 3b | 1.08 (0.99,1.18), 0.073 |

a: Adjusted for sex and age.

b: Adjusted for sex, age, infection site, AKI, intestine disease, invasive mechanical ventilation time, APACHEII score, PH, creatinine.
